# Phosphatic Food in Debility

**Published:** 1873-10

**Authors:** 


### PHOSPHATIC FOOD IN DEBILITY.

Dr. Routh, of London, gives among others the following instructive cases, in the *Medical Press and Circular.*

July 1. Rev. T. H. F., æt. about 60, has been a clergyman for many years, preaching with notes only, but lately has become confused whilst preaching, forgetting the thread ; seems also to have experienced lately want of power to grasp subjects. Recovers himself after a time, but the fear of this makes him very nervous; sleeps fairly, not troubled by dreams; lives in Cheshire, in a damp, cold neighborhood ; loss of memory occurs frequently at other times than when preaching ; no recollection, especially of names and figures ; urine normal, no sediment ; total loss of virile power ; no backache, but a creeping sensation up from the nape of the neck ; no loss of muscular power on either side ; eyesight weak ; no indigestion ; cannot digest lobster ; first sound of heart rather prolonged, especially at the base; bowels regular in London, more so than in the country. Ordered Parrish's food, oyster and other shell fish, excepting lobster. As his teeth are bad, use a small digestive sausage machine.

July 31. Greatly better. Had profited greatly from the treatment. The mental faculties much improved. States is not the same man. He was now ordered allotropic phosphorous, g. x. daily, after his dinner. My last account from this gentleman was that he had completely recovered.

Mrs. Y., æt. about 42, consulted me in November last for loss of mental power and strength. The catamenia had stopped twelve months, and she too had a large family, with small means, and was much worried by creditors. Her memory is very defective, indeed, gone; she can't remember anything, nor when she puts away any article of dress; When she has a good night she is rather better for a few hours and then the same state recurs. She is always worse if she has had her attention forcibly called to anything; is very restless at night, her feet being drawn up as if she was going to have a convulsion; is become shockingly bad-tempered; will become violent on the slightest contradiction; feels very anxious and unhappy; bowels open; tongue clean; no leucorrhœa at present, although five months back she used to have them copiously for two or three days, in lieu of the catamenia.

Ordered mustard to nape of neck; feet in hot water; half a drachm of bromide of potassium every night in water; sol. phosph. used m. x. *ter die.* A week after (November 12), was generally better, except that she had one bad day.

On the 19th she was better, but stated that she had taken the bromide very irregularly, finding she could sleep without it, and the head was much less giddy.

This patient I saw for several weeks after. The treatment was interrupted by a bilious attack, which obliged me to suspend the phosphorus; subsequently it was resumed. She is now greatly better; feels that the phosphorus acts as a sort of a tonic, or rather, as she expresses it, can't sleep without it. Memory greatly improved; some days not so good; but the intervals are longer, and generally her improvement is marked, and she is, in fact, convalescent.

